# Commentary: Methodological considerations in the interpretation of evidence on cannabis-based medicines for chronic neuropathic pain

**DOI:** 10.1186/s42238-026-00448-2

**Published:** 2026-05-27

**Authors:** Wellington Briques, Débora Maria Russiano Pereira, Aderbal Silva Aguiar Junior

**Affiliations:** 1 Institute for Education and Research (IEP) Remederi, São Paulo, SP Brazil; 2 Laboratory of Exercise Biology (LABIOEX), Department of Physical Therapy, Federal University of Santa Catarina (UFSC), Araranguá, SC Brazil; 3 University Library (Araranguá Campus), Federal University of Santa Catarina (UFSC), Araranguá, SC Brazil; 4 Laboratory of Exercise Biology, Federal University of Santa Catarina-UFSC, Araranguá, SC Brazil

**Keywords:** Cannabis-based medicines, Neuropathic pain, GRADE, Systematic reviews

## Abstract

Recent systematic reviews, including a Cochrane review, have concluded that the certainty of evidence supporting the use of cannabis-based medicines for chronic neuropathic pain remains low. While this overall conclusion is reasonable, its interpretation warrants careful scrutiny. In this commentary, key methodological and interpretative aspects relevant to the appraisal of this evidence base are examined, with particular attention to the application of the GRADE framework, clinical heterogeneity, outcome selection, and indirectness. It is proposed that inconsistent or insufficiently transparent application of methodological criteria may influence how results are interpreted and communicated, with potential implications for clinical decision-making. A cautious, consistent, and context-aware interpretation of the available evidence is essential to avoid both overstatement and undue dismissal of therapeutic effects in a complex and heterogeneous condition such as neuropathic pain.

## Background

Chronic neuropathic pain, defined as pain arising from a lesion or disease of the somatosensory nervous system, remains a prevalent and disabling condition, frequently refractory to available pharmacological treatments. Cannabis-based medicines have attracted increasing attention as potential therapeutic options, leading to an expanding body of randomized trials and systematic reviews. These reviews have consistently concluded that the certainty of evidence supporting clinical efficacy remains low, largely due to methodological limitations inherent to the available studies.

While such conclusions are broadly justified, the interpretation of low-certainty evidence in complex clinical conditions requires careful contextualization. Neuropathic pain trials are characterized by substantial clinical and methodological heterogeneity, challenges in outcome selection, and frequent reliance on short-term designs that may not fully capture clinically meaningful effects. In this context, the way methodological frameworks are applied and communicated may substantially influence the perceived balance between uncertainty and potential benefit.

This commentary examines key methodological and interpretative considerations relevant to the appraisal of cannabis-based medicines for chronic neuropathic pain. Rather than disputing the overall assessment of limited certainty, the focus is placed on how consistency, transparency, and clinical context in evidence evaluation may shape conclusions and their translation into clinical decision-making. This manuscript is intended as an independent methodological commentary, rather than as a direct response to the Cochrane review or a request for clarification from its authors.

## Main text

An updated Cochrane review recently evaluated the use of cannabis-based medicines for chronic neuropathic pain and concluded that the certainty of evidence remains low, with currently available trials insufficient to support strong clinical recommendations (Ateş et al. 2026). This overall assessment is broadly supported. The review represents a rigorous and methodologically robust synthesis, supported by a comprehensive search strategy and transparent application of the GRADE (Grading of Recommendations Assessment, Development and Evaluation) framework for rating certainty of evidence. However, the considerations presented here are not intended to alter this conclusion, but to inform how it is interpreted and communicated. Several aspects of the methodological assessment and interpretative framing merit closer examination, as they may influence how readers understand the strength, limitations, and implications of the findings.

The review appropriately identifies major limitations across the included trials, including short duration, small sample sizes, cross-over designs, and substantial clinical and pharmacological heterogeneity (Ateş et al. 2026). These limitations are clearly described and justify downgrading the certainty of evidence. The present considerations do not relate to the use of the GRADE framework itself, but rather to the consistency with which specific GRADE domains are applied across intervention groups informed by studies with broadly comparable designs and constraints. For example, several randomized trials informing THC-dominant interventions, such as smoked cannabis studies in neuropathic pain with crossover designs and limited sample sizes (as cited in the review, e.g., Abrams et al. 2007; Ware et al. 2010), share key methodological features with trials evaluating other cannabinoid formulations. Similarly, trials evaluating oromucosal nabiximols in neuropathic pain and multiple sclerosis populations (as cited in the review, e.g., Rog et al. 2005; Notcutt et al. 2004) frequently involve small samples, short follow-up periods, and clinically selected populations. While downgrading for domains such as imprecision or indirectness is appropriate, consistent application of these criteria across intervention categories informed by comparable evidence bases would benefit from clearer and more explicit justification (Guyatt et al. 2008).

Indirectness, as a specific GRADE domain, is given substantial weight as a driver of downgrading, primarily because several included trials enrolled clinically selected populations or focused on condition-specific outcomes (Ateş et al. 2026). This represents a valid consideration within the GRADE framework. However, the emphasis placed on indirectness may differ across intervention groups informed by trials with comparable clinical contexts. For example, studies of oromucosal nabiximols in multiple sclerosis populations (as cited in the review, e.g., Rog et al. 2005; Notcutt et al. 2004) are frequently downgraded due to population specificity, whereas trials of inhaled THC in neuropathic pain (e.g., Abrams et al. 2007; Ware et al. 2010), which also involve selected patient groups and controlled experimental conditions, raise similar considerations regarding generalizability. These differences may reflect variations in clinical context and outcome selection rather than clear differences in relevance. In this setting, a more explicit rationale for differential downgrading for indirectness across subgroups would enhance transparency and support consistent interpretation of the evidence (Guyatt et al. 2008).

Another consideration relates to the aggregation of pharmacologically distinct interventions under broad categories, such as THC-dominant, THC/CBD-balanced, or CBD-dominant products (Ateş et al. 2026). Inhaled herbal cannabis, oral synthetic cannabinoids, oromucosal sprays, low-dose oral cannabidiol, and topical preparations differ substantially in pharmacokinetics, bioavailability, tolerability, and expected clinical effects. Pooling such diverse interventions inevitably increases clinical and statistical heterogeneity and may obscure signals present only within specific formulations, routes of administration, or patient subgroups.

The review largely treats chronic neuropathic pain as a relatively homogeneous clinical entity. Contemporary pain research, however, recognizes substantial heterogeneity across phenotypes, including distinctions between peripheral and central mechanisms, sensory gain versus sensory loss profiles, and mixed nociplastic components (Baron et al. 2017). In the absence of phenotypic stratification, treatment effects linked to specific biological mechanisms may be diluted when mechanistically distinct conditions are analyzed together, thereby limiting interpretability.

The emphasis on dichotomous outcomes and strict thresholds for clinical relevance, such as number needed to treat values below 10, warrants careful contextualization (Ateş et al. 2026). Although a broad range of outcomes is considered in the review, the relative emphasis on dichotomous pain thresholds may influence the interpretation of clinically meaningful benefits. Even when stakeholder or patient perspectives are incorporated during review development, their influence on outcome prioritization is not always explicit in the final reporting. In chronic neuropathic pain, where responder rates are modest across most established pharmacological classes, clinically meaningful benefit may manifest as improvements in sleep, global patient-reported outcomes, or functional domains rather than large reductions in mean pain intensity alone (Dworkin et al. 2008). In this context, the absence of a clearly articulated hierarchy between patient-centered outcomes and disease-oriented pain metrics may contribute to underrecognition of benefits that are meaningful to patients but do not meet rigid binary thresholds.

Finally, safety signals associated with THC-containing products are clinically relevant and warrant careful consideration. Increased nervous system adverse events and higher discontinuation rates underscore the importance of careful patient selection, dose titration, and realistic treatment goals (Ateş et al. 2026). However, short-duration trials may overestimate early adverse effects and underestimate longer-term tolerability following titration, a limitation long recognized in chronic pain research (Moore et al. 2013).

In summary, the review demonstrates that the current evidence base for cannabis-based medicines in chronic neuropathic pain remains methodologically immature and insufficient to address key clinical questions regarding long-term efficacy and safety. It does not, however, establish definitive evidence of ineffectiveness. Greater consistency in the application of GRADE domains across comparable study groups, explicit acknowledgment of the heterogeneity inherent to neuropathic pain, and clearer contextualization of clinical relevance thresholds would enhance interpretability and reduce the risk of overgeneralization. As highlighted in previous high-quality syntheses of neuropathic pain pharmacotherapy, modest efficacy, substantial placebo responses, heterogeneous phenotypes, and persistent trial design limitations remain central challenges for the field (Finnerup et al. 2015). Until more robust and stratified data become available, a cautious approach to interpretation remains essential—not only in clinical recommendations, but also in how conclusions are communicated beyond their methodological context. This commentary is based on the published reporting of the review and does not involve reanalysis of the underlying data; accordingly, the considerations presented here should be interpreted as a methodological and interpretative perspective.

## Data Availability

No datasets were generated or analysed during the current study.
